# Personality disorders and cardiometabolic diseases: A Mendelian randomization study

**DOI:** 10.1097/MD.0000000000046702

**Published:** 2026-01-09

**Authors:** Wentao Yang, Zhaoqing Yang, Juemin Xi, Cheng Bian, Zian Cheng

**Affiliations:** a Department of Medical Genetics, Institute of Medical Biology, Chinese Academy of Medical Sciences & Peking Union Medical College, Kunming, Yunnan, People’s Republic of China; b National Research Institute for Family Planning, Chinese Academy of Medical Sciences & Peking Union Medical College, Beijing, People’s Republic of China.

**Keywords:** cardiometabolic diseases, Mendelian randomization, personality disorders

## Abstract

Observational studies have suggested the associations between personality disorders (PDs) and cardiometabolic diseases (CMDs), though these may be biased by unmeasured confounding, including lifestyle factors or reverse causality. Mendelian randomization (MR) provides a novel approach to address these limitations by using genetic variants as instrumental variables, thereby minimizing environmental confounding and enabling causal inference. This study leverages MR to systematically evaluate causal relationships across 9 clinically relevant PDs and 8 major CMDs, offering genetic evidence to clarify previously observed epidemiological associations. Data for 9 kinds of PDs (including anankastic personality disorder, anxious personality disorder, dependent personality disorder, dissocial personality disorder, emotionally unstable personality disorder, histrionic personality disorder, paranoid personality disorder, organic personality disorder, and schizoid personality disorder) were obtained from the FinnGen consortium R10 release and data for 8 kinds of CMDs (including myocardial infarction, atrial fibrillation, type 2 diabetes, hypertension, stroke, coronary atherosclerosis, ischemic stroke and peripheral artery disease) were obtained from UK biobank. Instrumental variables were selected using single-nucleotide polymorphisms meeting a genome-wide significance threshold (*P* < 5 × 10⁻⁶) and clumped (*R*^2^ < 0.001) to ensure independence. Primary causal estimates were generated via inverse-variance weighted regression, with sensitivity analyses MR-Egger, Cochran *Q* intercept analysis, and leave-one-out analysis to assess robustness. Genetically predicted organic personality disorder had a positive association with coronary atherosclerosis (OR = 1.001, 95% confidence interval, 1.000–1.002, *P* = .029). No other significant differences (*P* > .05) were found. Results were stable across sensitive analyses. Our findings contrast with prior observational studies, suggesting that reported associations may reflect residual confounding from environmental or behavioral risk factors, rather than biological causation. Future research should investigate behavioral or environmental mediators underlying these epidemiological correlations.

## 1. Introduction

Personality disorders (PDs), as defined by the International Classification of Diseases 10th Revision (ICD-10), represent enduring maladaptive patterns of cognition, emotional regulation, and behavior that cause significant functional impairment.^[1]^ With an estimated prevalence of 10% in the general population, PDs exhibit substantial clinical heterogeneity that poses challenges for epidemiological research.^[2]^ Previous research indicates that some PD often co-occur with unhealthy behaviors, increasing the risk factors associated with higher cardiometabolic risk factors, which were in turn associated with observed increases in diagnosis of cardiometabolic diseases (CMDs).^[3,4]^ Observational studies have suggested potential links between PDs and CMDs through behavioral pathways, with the National Epidemiologic Survey on Alcohol and Related Conditions study reporting a 32% increased cardiovascular risk associated with schizoid personality disorder (SCHIZPER) and the UK National Psychiatric Survey identifying borderline personality disorder (BPD, also known as emotionally unstable personality disorder) and SCHIZPER (OR = 1.29) as independent predictors of ischemic heart disease.^[5,6]^ However, observational studies examining associations between PDs and CMDs face inherent methodological limitations, including residual confounding from lifestyle factors and socioeconomic status, potential reverse causality, and diagnostic heterogeneity.

To overcome these methodological limitations, Mendelian randomization (MR), a genetic epidemiological approach that leverages single nucleotide polymorphisms (SNPs) from genome-wide association studies (GWAS) as instrumental variables (IVs) was employed. MR capitalizes on the biological principle that genetic variants are fixed at conception and remain unaffected by subsequent environmental exposures or disease processes, thereby minimizing confounding and reverse causation biases inherent in observational studies. This approach enables robust causal inference between exposures and outcomes while substantially reducing environmental confounding.^[7]^ In this study, a two-sample MR analysis using data from the FinnGen study and UK Biobank was conducted. These large-scale biobanks provide extensive sample sizes and comprehensive phenotypic data, enabling robust investigation of potential causal relationships between ICD-10 classified PDs and CMDs outcomes, including myocardial infarction, atrial fibrillation, type 2 diabetes, hypertension, stroke, coronary atherosclerosis, ischemic stroke, and peripheral artery disease. Using a population level analytical design, the observational correlations were transformed into genetically validated causal inferences through comprehensive evaluation of all 9 ICD-10 PDs and 8 CMDs. This methodological advance significantly extends beyond previous subtype-limited investigations, and bring insight into pathophysiological mechanisms between PDs and CMDs.

## 2. Methods

### 2.1. Study design

**Figure 1.**
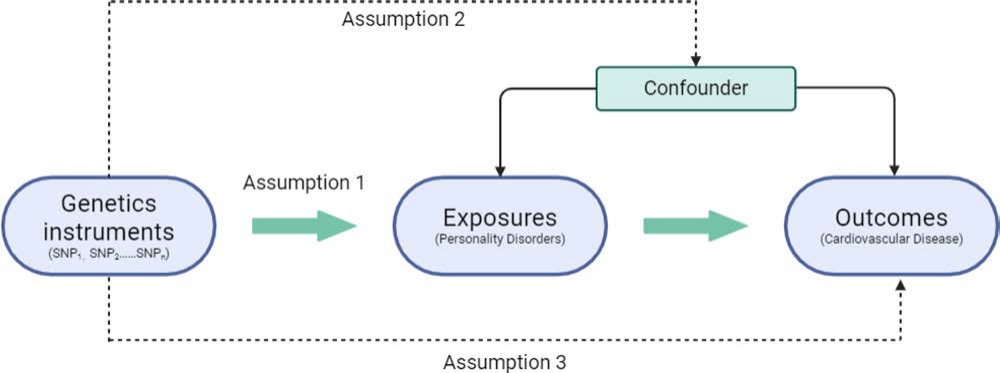
Overview and assumptions of the Mendelian randomization study design. Assumption 1: the IVs should be closely related to the risk factor of interest; assumption 2: the IVs should not be associated with potential confounders, and assumption 3: the IVs should affect the risk of outcome only through risk factors and not through other alternative pathways. IV = instrumental variable.

This research design employed existing GWAS summary statistics from Finngen consortium and UK biobank. The use of data from these previously approved and published analyses is considered methodologically appropriate as this study itself does not directly engage human subjects. We only reanalyzed, summarized, and pooled data that was already available, therefore no additional ethical approval or informed consent was needed. MR estimates causal effects by leveraging genetic variants as IVs, which are naturally randomized at conception and satisfy key assumptions: strong association with the exposure, independence from confounders, and exclusive influence on the outcome through the exposure (Fig. 1).^[8,9]^ By quantifying the genetic associations with both exposure and outcome, MR derives the causal effect of exposure on outcome, mimicking natural randomized experiments to avoid confounding and reverse causality in observational studies.

### 2.2. GWAS on PDs

Data for PDs were obtained from the FinnGen consortium R10 (University of Helsinki, Helsinki, Finland) release and cases were defined by the codes of the ICD-10 (Data S1, Supplemental Digital Content, https://links.lww.com/MD/Q953). The FinnGen study is an ongoing national cohort study launched in 2017, combining genetic data from the Finnish Biobank and health record data from the Finnish Health Registry. Among the existing PDs, we have selected 9 personality types that have clear definitions and diagnostic criteria, including anankastic personality disorder, anxious personality disorder, dependent personality disorder, dissocial personality disorder, emotionally unstable personality disorder, histrionic personality disorder, paranoid personality disorder, organic personality disorder (OPD), and SCHIZPER. Information of exposure GWAS data sets were listed in Table 1, quoted from FinnGen Consortium. More detailed information about FinnGen can be found on their official website (https://www.finngen.fi/en).

**Table 1:** Exposure GWAS data sets.

Study	Phenotype	Controls	Case	ICD-10 description
FinnGen R10	Anankastic personality disorder (ANAPER)	400,619	1147	**F60.5** Personality disorder characterized by feelings of doubt, perfectionism, excessive conscientiousness, checking and preoccupation with details, stubbornness, caution, and rigidity. There may be insistent and unwelcome thoughts or impulses that do not attain the severity of an obsessive-compulsive disorder.
FinnGen R10	Anxious personality disorder (ANXPER)	400,619	602	**F60.6** Personality disorder characterized by feelings of tension and apprehension, insecurity and inferiority. There is a continuous yearning to be liked and accepted, a hypersensitivity to rejection and criticism with restricted personal attachments, and a tendency to avoid certain activities by habitual exaggeration of the potential dangers or risks in everyday situations.
FinnGen R10	Dependent personality disorder (DEPPER)	400,619	656	**F60.7** Personality disorder characterized by pervasive passive reliance on other people to make one’s major and minor life decisions, great fear of abandonment, feelings of helplessness and incompetence, passive compliance with the wishes of elders and others, and a weak response to the demands of daily life. Lack of vigor may show itself in the intellectual or emotional spheres; there is often a tendency to transfer responsibility to others.
FinnGen R10	Dissocial personality disorder (DISPER)	400,619	507	**F60.2** Personality disorder characterized by disregard for social obligations, and callous unconcern for the feelings of others. There is gross disparity between behavior and the prevailing social norms. Behavior is not readily modifiable by adverse experience, including punishment. There is a low tolerance to frustration and a low threshold for discharge of aggression, including violence; there is a tendency to blame others, or to offer plausible rationalizations for the behavior bringing the patient into conflict with society.
FinnGen R10	Emotionally unstable personality disorder (EMOPER)	400,619	4551	**F60.3** Personality disorder characterized by a definite tendency to act impulsively and without consideration of the consequences; the mood is unpredictable and capricious. There is a liability to outbursts of emotion and an incapacity to control the behavioral explosions. There is a tendency to quarrelsome behavior and to conflicts with others, especially when impulsive acts are thwarted or censored. Two types may be distinguished: the impulsive type, characterized predominantly by emotional instability and lack of impulse control, and the borderline type, characterized in addition by disturbances in self-image, aims, and internal preferences, by chronic feelings of emptiness, by intense and unstable interpersonal relationships, and by a tendency to self-destructive behavior, including suicide gestures and attempts.
FinnGen R10	Histrionic personality disorder (HISPER)	400,619	148	**F60.4** Personality disorder characterized by shallow and labile affectivity, self-dramatization, theatricality, exaggerated expression of emotions, suggestibility, egocentricity, self-indulgence, lack of consideration for others, easily hurt feelings, and continuous seeking for appreciation, excitement and attention.
FinnGen R10	Paranoid personality disorder (PARAPER)	400,619	608	**F60.0** Personality disorder characterized by excessive sensitivity to setbacks, unforgiveness of insults; suspiciousness and a tendency to distort experience by misconstruing the neutral or friendly actions of others as hostile or contemptuous; recurrent suspicions, without justification, regarding the sexual fidelity of the spouse or sexual partner; and a combative and tenacious sense of personal rights. There may be excessive self-importance, and there is often excessive self-reference.
FinnGen R10	Organic Personality Disorder (OPD)	388,560	463	**F07** Alteration of personality and behavior can be a residual or concomitant disorder of brain disease, damage or dysfunction.
FinnGen R10	Schizoid personality disorder (SCHIZPER)	400,619	580	**F60.1** Personality disorder characterized by withdrawal from affectional, social and other contacts with preference for fantasy, solitary activities, and introspection. There is a limited capacity to express feelings and to experience pleasure.

GWAS = genome-wide association.

### 2.3. GWAS on CMDs

Summary statistics for GWAS on CMDs were obtained from UK Biobank. The UK Biobank cohort is a prospective general population cohort that recruited 502,628 participants aged 40 to 70 from the general population between 2006 and 2010.^[10]^ The participants’ clinical data are derived from hospital medical records and self-reports. The diagnoses of CMDs were coded according to ICD-10. Myocardial infarction, atrial fibrillation, type 2 diabetes, hypertension, stroke, coronary atherosclerosis, ischemic stroke, and peripheral artery disease were chosen as outcomes. The demographic profiles of outcomes involved in this study are summarized in Table 2.

**Table 2:** Outcome GWAS data sets.

Study	Phenotype	Control	Case	Authors
UK Biobank	Myocardial infarction	473,517	11,081	Dönertaş HM
UK Biobank	Atrial fibrillation	481,061	3537	Dönertaş HM
BioBank Japan, UK Biobank, FinnGen	Type 2 diabetes	451,248	38,841	Sakaue S
UK Biobank	Hypertension	354,689	129,909	Dönertaş HM
UK Biobank	Stroke	477,673	6925	Dönertaş HM
UK Biobank	Coronary atherosclerosis	346,860	14,334	Neale lab
BioBank Japan, UK Biobank, FinnGen	Ischemic stroke	472,192	11,929	Sakaue S
BioBank Japan, UK Biobank, FinnGen	Peripheral artery disease	475,964	7114	Sakaue S

GWAS = genome-wide association.

### 2.4. Selection of IVs

All SNPs that strongly and independently (*R*^2^ < 0.001) predict exposures at genome-wide significance (*P* < 5 × 10^–8^) were used. To acquire more independent SNPs with genome-wide significance, a higher cutoff (*P* < 5 × 10^–6^) was also used for obtaining SNPs predicting the PDs. To ensure the independence among SNPs, single nucleotide polymorphisms were filtered using the linkage disequilibrium score pruning method, a genomic window of 10,000 kb was set. The instrumental strength for the SNP-exposure association was calculated as the average of SNP-specific *F*-statistics approximated by the square of the beta divided by the variance for the SNP-exposure association, a value >10 was considered sufficient, and the results of the MR analysis could avoid being affected by weak-tool bias.^[11]^ For specific code, refer to Data S2, Supplemental Digital Content, https://links.lww.com/MD/Q953.

### 2.5. Two-sample MR analysis

All analyses were performed using R (v4.3.2) statistical software (the R Foundation for Statistical Computing, Vienna, Austria). MR analyses were performed using the R-based package “TwoSampleMR.”

Weighted multivariable-adjusted logistic regression was used to calculate the odds ratio (OR) with 95% confidence interval (CI), and *P*-value < 0.05 was considered statistically significant. When the OR value is >1, it indicates a positive correlation between exposure and outcome, which means that increasing exposure factors leads to an increase in risk factors for outcomes, implying that the exposure may be a risk factor for outcomes. When the OR value is <1, it indicates a negative correlation between exposure and outcomes, which means that increasing exposure factors will result in a decrease in the risk factor for outcomes, implying that exposure may be a protective factor for outcomes. Inverse variance weighted (IVW) was primarily used to assess the association of genetically predicted PDs and CMDs, which were calculated as the weighted mean of the individual ratio estimates, with weights equaling the inverse-variances of the ratio estimates.^[12]^ IVW method has become the primary analytical approach in MR studies due to its high efficiency, robustness, and compatibility with multiple instrumental variables. The specific code and R packages can be found in the Data S3 and Data S4, Supplemental Digital Content, https://links.lww.com/MD/Q953. We assumed that all of the IVs were valid and could provide an imprecise estimate if the MR assumptions were not met.

### 2.6. Sensitivity analyses

We conducted comprehensive sensitivity analyses to evaluate the robustness of our MR estimates. MR-Egger regression was employed to assess directional pleiotropy, where its intercept term quantifies the average horizontal pleiotropic effect across SNPs, a statistically significant intercept (*P* < 0.05) would indicate potential bias in our primary IVW estimates.^[13]^ (Data S5, Supplemental Digital Content, https://links.lww.com/MD/Q953) To identify influential variants, we performed leave-one-out analyses by iteratively excluding individual SNPs and visualizing results through forest plots, ensuring no single genetic instrument disproportionately drove our findings (Data S6 and Data S7, Supplemental Digital Content, https://links.lww.com/MD/Q953) Cochran *Q* test examined heterogeneity among SNP-specific estimates, with *P* > 0.05 supporting the use of fixed-effects IVW indicating homogeneous effects, and *P* ≤ 0.05 warranting random effects IVW indicating an accommodating between SNP variability (Data S8, Supplemental Digital Content, https://links.lww.com/MD/Q953) These complementary approaches can collectively validate that if our results were not substantially affected by pleiotropy, outlier SNPs, or heterogeneous effects.

## 3. Results

### 3.1. Characteristics of the selected SNPs

IVs associated with 9 kinds of PDs were extracted from the GWAS at a higher cutoff (*P* < 5 × 10^−6^) to obtain more independent SNPs at genome-wide significance. To ensure accuracy, linkage disequilibrium analysis was performed to eliminate any potential chain ambiguity (*r*^2^ < 0.001, 10,000-kb). A total of 63 SNPs were selected. The screened SNPs were included in further analyses in which *F* values were calculated. All *F* values were >20 and was deemed satisfactory as such reduces concern regarding potential issues of bias arising from a “weak instrument effect” or the potential impact due to noise in estimation. All SNPs used in the study, along with their basic summary statistics including average beta effect-sizes *F* statistics, *P* values associated with the main genetic IVs for all analyses performed are listed in Table 3.

**Table 3:** IVs selected.

PD	SNP	Gene	EA	Pval	Beta	SE	Fval
ANAPER	rs4927063	CDCP2	T	1.86E‐06	‐0.22	0.05	22.74
	rs72737195	IRX1	C	3.50E‐06	0.55	0.12	21.52
	rs146405735	DNAH5	C	1.76E‐06	0.46	0.10	22.84
	rs6461853	NPVF	G	1.82E‐07	0.22	0.04	27.21
	rs41283536	ANK3	G	5.23E‐07	0.26	0.21	25.18
	rs35560772	BRMS1L	T	2.12E‐07	‐0.39	0.07	26.93
ANXPER	rs7527268	CAMTA1	C	1.38E‐06	‐0.29	0.06	23.30
	rs12141028	DENND1B	A	3.31E‐06	‐0.39	0.08	21.63
	rs2420401	GOLIM4	G	2.89E‐06	‐0.83	0.18	21.89
	rs12054583	FGF5	G	4.62E‐06	0.26	0.06	20.99
	rs59384743	CCSER1	T	6.97E‐07	0.70	0.14	24.62
	rs4704968	EBF1	G	4.98E‐06	0.28	0.06	20.85
	rs11758932	AIG1	C	4.39E‐06	0.32	0.07	21.09
	rs6651477	PSD3	G	1.86E‐06	3.61	0.76	22.74
	rs7870396	VLDLR	T	6.01E‐07	‐0.28	0.06	24.91
	rs61845064	ARMC4	C	2.76E‐06	2.07	0.44	21.97
	rs34440054	ITGA5	A	3.18E‐06	1.30	0.28	21.70
DEPPER	rs72696649	CLCN3	A	8.19E‐07	0.29	0.06	24.31
	rs213958	CFTR	A	4.81E‐07	0.30	0.06	25.34
	rs28875315	MGAM2	A	4.62E‐06	‐0.55	0.12	20.99
	rs7039637	TLR4	C	5.60E‐07	‐0.42	0.08	25.05
	rs11187172	HHEX	A	3.70E‐07	0.69	0.14	25.85
	rs117236352	HSD17B12	A	6.71E‐07	0.84	0.17	24.70
	rs75109136	PSMD7	T	4.42E‐06	0.55	0.12	21.07
	rs72806072	RPH3AL	C	4.91E‐06	0.28	0.06	20.87
DISPER	rs57972714	SEC16B	T	1.84E‐06	0.32	0.07	22.76
	rs142398905	LARP1B	T	1.34E‐06	0.92	0.19	23.37
	rs9943806	LMO3	G	4.60E‐06	0.38	0.08	21.00
EMOPER	rs61772073	ERRFI1	C	8.69E‐07	0.45	0.09	24.20
	rs55723474	TSPAN2	T	4.91E‐06	0.11	0.02	20.87
	rs7563730	RND3	C	2.98E‐06	‐0.25	0.05	21.83
	rs79452989	PKP4	A	3.36E‐06	0.20	0.04	21.60
	rs143297171	LIMD1	T	1.27E‐06	0.28	0.06	23.46
	rs2030892	ZSWIM6	G	1.11E‐07	‐0.12	0.02	28.18
	rs13185476	SGCD	G	1.03E‐07	0.14	0.03	28.32
	rs12164106	DNAH11	T	4.17E‐06	0.18	0.04	21.19
	rs7802800	LHFPL3	T	1.64E‐07	0.12	0.02	27.42
	rs59526505	KCNQ3	A	3.74E‐06	‐0.13	0.03	21.39
	rs113282715	CNTLN	T	2.63E‐06	0.17	0.04	22.07
	rs138392998	PSAT1	G	3.50E‐07	‐0.52	0.10	25.95
	rs78503173	OLFM1	T	3.96E‐06	0.19	0.04	21.28
	rs145747812	MORN4	G	5.64E‐07	0.31	0.06	25.03
	rs7393175	SORCS1	C	1.37E‐06	0.14	0.03	23.33
	rs4756784	SPON1	A	4.76E‐06	0.11	0.02	20.93
	rs637402	PCDH17	C	1.77E-07	0.15	0.03	27.27
	rs12717560	ELMSAN1	G	2.32E‐06	‐0.11	0.02	22.31
	rs148645962	CDC42BPB	T	2.93E‐06	‐0.39	0.08	21.86
	rs62196661	BMP2	A	1.86E‐07	0.13	0.03	27.17
	rs111412763	CRYBB2	G	3.43E‐06	‐0.20	0.04	21.56
HISPER	rs62355808	TRIM60	A	3.85E‐06	0.50	0.11	21.34
	rs191956392	RAB19	T	2.94E‐06	1.34	0.29	21.86
	rs117391983	FER1L6	T	3.53E‐06	0.89	0.19	21.50
PARAPER	rs10022387	CPEB2	T	3.41E‐06	6.06	1.31	21.57
	rs149440618	CDH18	C	2.16E‐06	0.31	0.07	22.45
	rs9790941	ADAMTS12	A	1.63E‐06	‐0.35	0.07	22.99
	rs12533912	ARL4A	A	4.93E‐06	‐0.52	0.11	20.87
	rs113813075	MGMT	A	4.43E‐06	0.67	0.15	21.07
	rs7307682	CCDC91	G	3.65E‐06	‐0.45	0.10	21.44
	rs72835659	JUP	A	4.67E‐06	0.36	0.08	20.97
OPD	rs3741863	PPM1H	G	6.28E‐07	0.69	0.14	24.82
	rs67661214	FLRT2	C	9.74E‐07	0.76	0.15	23.98
SCHIZPER	rs79399881	COL6A5	C	3.95E‐07	2.74	0.54	25.72
	rs117256068	CNTN5	A	5.54E‐07	1.61	0.32	25.07

ANXPER = anxious personality disorder, DEPPER = dependent personality disorder, DISPER = dissocial personality disorder, EMOPER = emotionally unstable personality disorder, HISPER = histrionic personality disorder, IV = instrumental variable, OPD = organic personality disorder, PARAPER = paranoid personality disorder, SCHIZPER = schizoid personality disorder, SNP = single nucleotide polymorphism.

### 3.2. Two-sample MR analysis of PDs and CMDs

**Figure 2.**
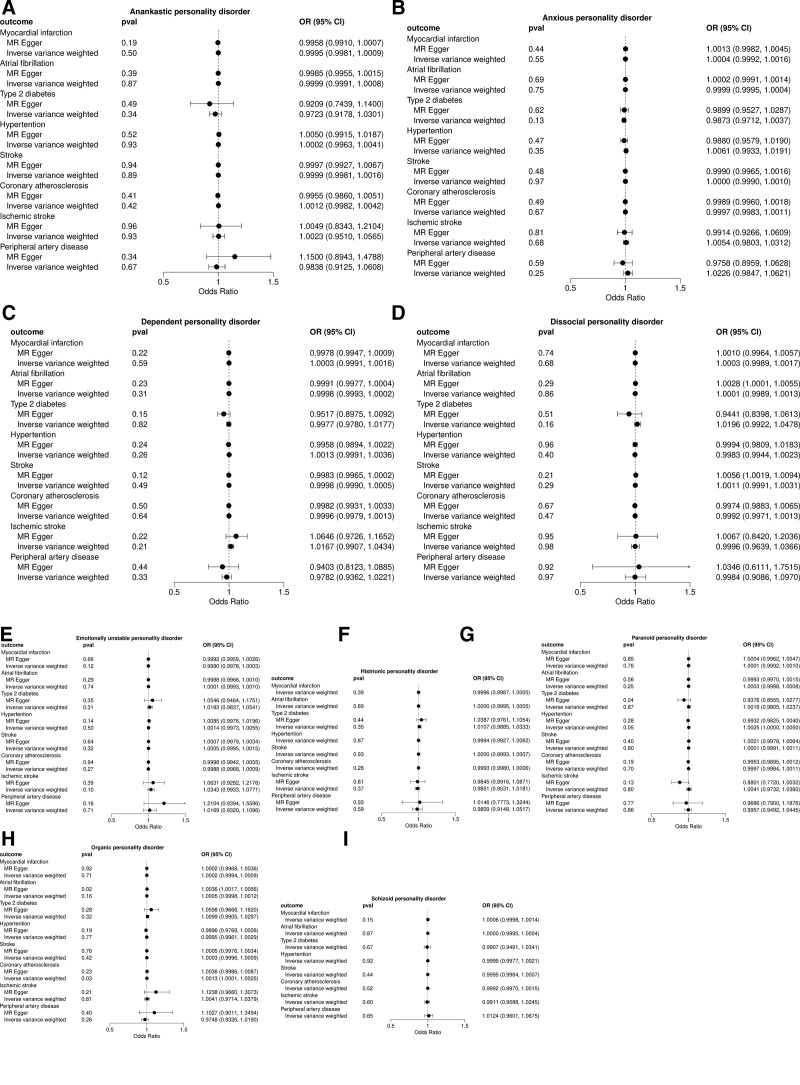
Forest plot of MR result. (A) Result for Anankastic personality disorder, (B) result for anxious personality disorder, (C) result for dependent personality disorder, (D) result for dissocial personality disorder, (E) result for emotionally unstable personality disorder, (F) result for histrionic personality disorder, (G) result for paranoid personality disorder, (H) result for organic personality disorder, (I) Result for schizoid personality disorder. MR = Mendelian randomization.

MR results are shown in Figure 2. According to IVW results, OPD showed a positive association with coronary atherosclerosis, indicating that the presence of OPD would increase the risk of coronary atherosclerosis (OR = 1.001, 95% CI, 1.000–1.002, *P* = 0.029). Other results indicate no significant difference (*P* > .05).

### 3.3. Sensitivity analyses

**Figure 3.**
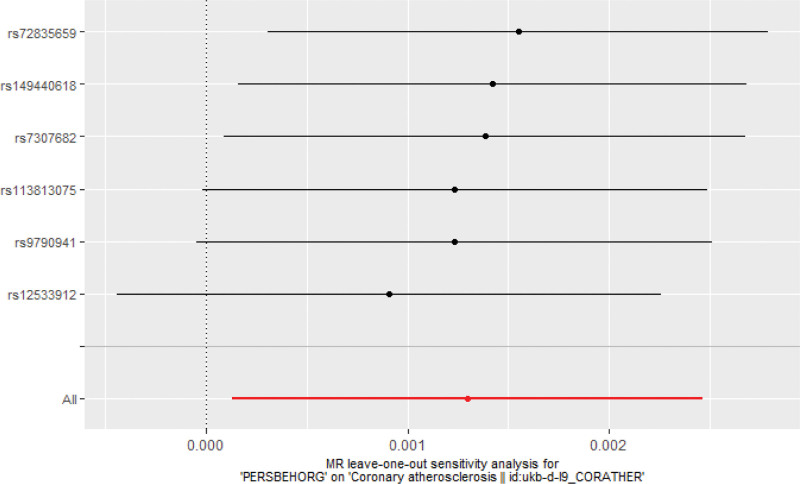
Leave-one-out sensitive analysis for OPD on coronary atherosclerosis. OPD = organic personality disorder.

This study introduced a variety of strategies for doing sensitivity analyses. Table 4 shows the results of Cochran *Q* test for heterogeneity, with *P* value > 0.05 indicating no heterogeneity between SNPs. Therefore, fixed-effects IVW method was chosen as the main analytical method in this study. Furthermore, MR-Egger intercept analysis suggested an absence of horizontal pleiotropy (Fig. 2). In addition, the leave-one-out study (Fig. 3) demonstrated that no single SNP was responsible for the putative causal relationship between PDs and CMD risk.

**Table 4:** Results of Cochran *Q* test.

Exposure	Outcome	Method	*Q*	*Q*_df	*Q*_pval
ANAPER	Myocardial infarction	MR Egger	0.63	3	0.89
ANAPER	Myocardial infarction	IVW	3.04	4	0.55
ANAPER	Atrial fibrillation	MR Egger	3.37	3	0.34
ANAPER	Atrial fibrillation	IVW	4.48	4	0.34
ANAPER	Type 2 diabetes	MR Egger	12.53	4	0.01
ANAPER	Type 2 diabetes	IVW	13.38	5	0.02
ANAPER	Hypertension	MR Egger	1.68	3	0.64
ANAPER	Hypertension	IVW	2.21	4	0.70
ANAPER	Stroke	MR Egger	9.60	3	0.02
ANAPER	Stroke	IVW	9.61	4	0.05
ANAPER	Coronary atherosclerosis	MR Egger	7.55	4	0.11
ANAPER	Coronary atherosclerosis	IVW	10.41	5	0.06
ANAPER	Ischemic stroke	MR Egger	2.33	4	0.67
ANAPER	Ischemic stroke	IVW	2.33	5	0.80
ANAPER	Peripheral artery disease	MR Egger	0.90	4	0.92
ANAPER	Peripheral artery disease	IVW	2.52	5	0.77
ANXPER	Myocardial infarction	MR Egger	15.80	6	0.01
ANXPER	Myocardial infarction	IVW	16.89	7	0.02
ANXPER	Atrial fibrillation	MR Egger	6.28	6	0.39
ANXPER	Atrial fibrillation	IVW	6.65	7	0.47
ANXPER	Type 2 diabetes	MR Egger	3.30	7	0.86
ANXPER	Type 2 diabetes	IVW	3.32	8	0.91
ANXPER	Hypertension	MR Egger	196.11	6	0.00
ANXPER	Hypertension	IVW	247.70	7	0.00
ANXPER	Stroke	MR Egger	15.97	6	0.01
ANXPER	Stroke	IVW	17.83	7	0.01
ANXPER	Coronary atherosclerosis	MR Egger	14.20	7	0.05
ANXPER	Coronary atherosclerosis	IVW	14.93	8	0.06
ANXPER	Ischemic stroke	MR Egger	9.70	7	0.21
ANXPER	Ischemic stroke	IVW	9.97	8	0.27
ANXPER	Peripheral artery disease	MR Egger	6.80	7	0.45
ANXPER	Peripheral artery disease	IVW	8.23	8	0.41
DEPPER	Myocardial infarction	MR Egger	10.61	6	0.10
DEPPER	Myocardial infarction	IVW	15.76	7	0.03
DEPPER	Atrial fibrillation	MR Egger	1.55	6	0.96
DEPPER	Atrial fibrillation	IVW	2.68	7	0.91
DEPPER	Type 2 diabetes	MR Egger	5.67	6	0.46
DEPPER	Type 2 diabetes	IVW	8.41	7	0.30
DEPPER	Hypertension	MR Egger	1.27	6	0.97
DEPPER	Hypertension	IVW	4.52	7	0.72
DEPPER	Stroke	MR Egger	5.58	6	0.47
DEPPER	Stroke	IVW	8.27	7	0.31
DEPPER	Coronary atherosclerosis	MR Egger	13.00	6	0.04
DEPPER	Coronary atherosclerosis	IVW	13.75	7	0.06
DEPPER	Ischemic stroke	MR Egger	1.45	6	0.96
DEPPER	Ischemic stroke	IVW	2.53	7	0.92
DEPPER	Peripheral artery disease	MR Egger	7.67	6	0.26
DEPPER	Peripheral artery disease	IVW	8.07	7	0.33
DISPER	Myocardial infarction	MR Egger	0.39	1	0.53
DISPER	Myocardial infarction	IVW	0.49	2	0.78
DISPER	Atrial fibrillation	MR Egger	0.24	1	0.63
DISPER	Atrial fibrillation	IVW	4.48	2	0.11
DISPER	Type 2 diabetes	MR Egger	0.16	1	0.69
DISPER	Type 2 diabetes	IVW	1.91	2	0.38
DISPER	Hypertension	MR Egger	2.15	1	0.14
DISPER	Hypertension	IVW	2.18	2	0.34
DISPER	Stroke	MR Egger	0.43	1	0.51
DISPER	Stroke	IVW	6.80	2	0.03
DISPER	Coronary atherosclerosis	MR Egger	1.76	1	0.18
DISPER	Coronary atherosclerosis	IVW	2.07	2	0.36
DISPER	Ischemic stroke	MR Egger	0.08	1	0.78
DISPER	Ischemic stroke	IVW	0.09	2	0.96
DISPER	Peripheral artery disease	MR Egger	4.63	1	0.03
DISPER	Peripheral artery disease	IVW	4.72	2	0.09
EMOPER	Myocardial infarction	MR Egger	16.73	18	0.54
EMOPER	Myocardial infarction	IVW	16.75	19	0.61
EMOPER	Atrial fibrillation	MR Egger	22.97	18	0.19
EMOPER	Atrial fibrillation	IVW	25.13	19	0.16
EMOPER	Type 2 diabetes	MR Egger	27.73	19	0.09
EMOPER	Type 2 diabetes	IVW	28.39	20	0.10
EMOPER	Hypertension	MR Egger	24.47	18	0.14
EMOPER	Hypertension	IVW	27.01	19	0.10
EMOPER	Stroke	MR Egger	11.48	18	0.87
EMOPER	Stroke	IVW	11.49	19	0.91
EMOPER	Coronary atherosclerosis	MR Egger	20.61	17	0.24
EMOPER	Coronary atherosclerosis	IVW	20.77	18	0.29
EMOPER	Ischemic stroke	MR Egger	18.78	19	0.47
EMOPER	Ischemic stroke	IVW	18.95	20	0.53
EMOPER	Peripheral artery disease	MR Egger	33.88	19	0.02
EMOPER	Peripheral artery disease	IVW	37.53	20	0.01
HISPER	Myocardial infarction	IVW	0.00	1	0.95
HISPER	Atrial fibrillation	IVW	0.03	1	0.86
HISPER	Type 2 diabetes	MR Egger	0.22	1	0.64
HISPER	Type 2 diabetes	IVW	1.08	2	0.58
HISPER	Hypertension	IVW	7.58	1	0.01
HISPER	Stroke	IVW	0.03	1	0.86
HISPER	Coronary atherosclerosis	IVW	0.00	1	0.96
HISPER	Ischemic stroke	MR Egger	0.33	1	0.56
HISPER	Ischemic stroke	IVW	0.33	2	0.85
HISPER	Peripheral artery disease	MR Egger	4.02	1	0.05
HISPER	Peripheral artery disease	IVW	4.30	2	0.12
PARAPER	Myocardial infarction	MR Egger	4.69	4	0.32
PARAPER	Myocardial infarction	IVW	4.71	5	0.45
PARAPER	Atrial fibrillation	MR Egger	4.11	4	0.39
PARAPER	Atrial fibrillation	IVW	4.99	5	0.42
PARAPER	Type 2 diabetes	MR Egger	2.21	4	0.70
PARAPER	Type 2 diabetes	IVW	4.34	5	0.50
PARAPER	Hypertension	MR Egger	0.41	4	0.98
PARAPER	Hypertension	IVW	3.41	5	0.64
PARAPER	Stroke	MR Egger	7.63	4	0.11
PARAPER	Stroke	IVW	9.19	5	0.10
PARAPER	Coronary atherosclerosis	MR Egger	1.50	4	0.83
PARAPER	Coronary atherosclerosis	IVW	3.81	5	0.58
PARAPER	Ischemic stroke	MR Egger	1.16	4	0.88
PARAPER	Ischemic stroke	IVW	5.28	5	0.38
PARAPER	Peripheral artery disease	MR Egger	1.93	4	0.75
PARAPER	Peripheral artery disease	IVW	2.00	5	0.85
OPD	Myocardial infarction	MR Egger	2.70	4	0.61
OPD	Myocardial infarction	IVW	2.70	5	0.75
OPD	Atrial fibrillation	MR Egger	1.18	4	0.88
OPD	Atrial fibrillation	IVW	11.62	5	0.04
OPD	Type 2 diabetes	MR Egger	2.47	4	0.65
OPD	Type 2 diabetes	IVW	3.57	5	0.61
OPD	Hypertension	MR Egger	7.79	4	0.10
OPD	Hypertension	IVW	12.41	5	0.03
OPD	Stroke	MR Egger	4.78	4	0.31
OPD	Stroke	IVW	4.81	5	0.44
OPD	Coronary atherosclerosis	MR Egger	1.67	4	0.80
OPD	Coronary atherosclerosis	IVW	2.56	5	0.77
OPD	Ischemic stroke	MR Egger	4.57	4	0.33
OPD	Ischemic stroke	IVW	7.10	5	0.21
OPD	Peripheral artery disease	MR Egger	3.80	4	0.43
OPD	Peripheral artery disease	IVW	5.30	5	0.38
SCHIZPER	Myocardial infarction	IVW	0.13	1	0.72
SCHIZPER	Atrial fibrillation	IVW	0.05	1	0.82
SCHIZPER	Type 2 diabetes	IVW	3.51	1	0.06
SCHIZPER	Hypertension	IVW	0.55	1	0.46
SCHIZPER	Stroke	IVW	3.26	1	0.07
SCHIZPER	Coronary atherosclerosis	IVW	3.52	1	0.06
SCHIZPER	Ischemic stroke	IVW	0.03	1	0.86
SCHIZPER	Peripheral artery disease	IVW	0.15	1	0.70

ANAPER = anankastic personality disorder, ANXPER = anxious personality disorder, DEPPER = dependent personality disorder, DISPER = dissocial personality disorder, EMOPER = emotionally unstable personality disorder, HISPER = histrionic personality disorder, IVW = inverse variance weighted, MR = Mendelian randomization, OPD = organic personality disorder, PARAPER = paranoid personality disorder, SCHIZPER = schizoid personality disorder.

## 4. Discussion

The study aimed at estimating a possible genetic association using MR analysis on available GWAS data of 9 PD phenotypes and 8 types of CMD. Based on all performed sensitivity assessments and strict instrumental selection criteria described above, only OPD was positively associated to an increase in coronary artery disease (OR = 1.001, 95% CI, 1.000–1.002, *P* = 0.029). The remaining PD–CMD associations were nonsignificant. These predominantly null results may reflect true biological independence, though methodological limitations must be considered, including limited statistical power to detect small effect sizes given current sample sizes or incomplete coverage of PD genetic architectures in existing GWAS.

According to ICD-10, OPD refers to personality or behavioral disorders resulting from residuals or concomitant disorders due to brain disease, injury, or dysfunction. Patients with OPD exhibit significant changes in habitual patterns compared to their behavior before the illness, particularly affecting emotions, needs, and impulses.^[14]^ The etiology is diverse, primarily secondary to primary brain injury or dysfunction, including stroke, Parkinson disease, multiple sclerosis, etc. There is epidemiological evidence that stroke and coronary atherosclerosis phenotypes have significant rates of co-occurrence with similar risk factors.^[3,15,16]^ However, since the selected population is mainly based on epidemiological statistics, there may be factors of confounding etiology that affect these 2 diseases; the SNPs we have selected may not be able to fully explain these processes. Although this method still cannot completely rule out certain pleiotropic mechanisms. Stroke and coronary atherosclerosis both fall under atherosclerotic cardiovascular disease and since atherosclerosis is related to genetics, it is hard to consider that there is a genetic relationship between OPD and coronary atherosclerosis.

Clinical associations between PDs and CMDs frequently reported in observational studies may primarily arise from mediating factors (such as behavioral patterns, environmental influences, or shared comorbidities) rather than direct genetic connections. This interpretation aligns with the well-documented phenotypic heterogeneity across PD and CMD subtypes. In a cross-sectional study involving 12,792 adults, researchers found that PD patients were more likely to have a stroke or ischemic heart disease.^[6]^ A large-scale study in Sweden, involving 1,107,524 people, including 9185 (0.83%) with PD, showed that patients with PD suffered from cardiovascular disease 1.4 times higher than those without PD.^[17]^

A study showed that adolescents with PDs were more likely obese by the age of 33.^[18]^ Also, a cross-sectional study found that the incidence of metabolic syndrome in BPD patients was twice as high as control group, independent of the body mass index.^[19]^ In a follow-up assessment, participants with PDs were more likely to lack regular exercise than their peers (OR = 1.35, 95% CI = 1.09–1.67).^[20]^ Also, some PD individuals may engage excessively in unhealthy behaviors, such as smoking, excessive alcohol, and reduced physical activity.^[21]^ Among those above, metabolic abnormalities, smoking, excessive alcohol consumption, and lack of exercise were well-known risk factors for CMDs.^[22]^

Psychosocial factors may also play a role as mediators.^[23]^ PD patients may experience negative emotions and social inhibition. A previous study surveyed and statistically analyzed a group of diabetic patients, and researchers found that the diabetes distress and social isolation experienced by patients with PDs may have an indirect effect on glycated hemoglobin and health-related quality of life.^[24]^ This is also one of the factors that could lead to an increased risk of CMDs or indicate a poor prognosis. Furthermore, more follow-up studies are required to establish the relationship between PDs and CMDs, as well as the mediating factors.

Additionally, the classification and definition of different types of PDs vary among different disease classification directories (such as ICD-10 and DSM-5), which may lead to varying understandings and analyses of PDs in different studies. Though according to earlier study, some PDs in ICD-10 and DSM-5 catalogs seems paired, there are still non-paired diagnoses.^[25]^ Therefore, a unified classification and definition system will be beneficial in order to better understand and analyze the relationship between PDs and CMDs, which may also contribute to a more accurate identification and intervention with personality traits that may increase the risk of CMDs.

In this study, we excluded certain types of PDs that lack detailed descriptions or clear diagnostic criteria, including mixed personality disorder, other and unspecified personality disorders in adults, and other specified and unspecified personality disorders, because their symptoms vary widely and are difficult to analyze across different studies, hence the results may be driven by certain patient populations. However, many patients fit under these categories. Mixed personality disorder, for instance, is one of the common types of PD, affecting approximately 3% to 6% of adults.^[26]^ Therefore, additional research is required after a more complete taxonomy of these categories.

This study provides notable advancements in understanding the potential genetic links between PDs and CMDs. Our analysis represents the most comprehensive MR investigation to date, encompassing all 9 ICD-10 classified PDs and 8 major CMDs (a significant expansion beyond prior studies that typically examined only limited PD subtypes). This broad phenotypic coverage enhances the generalizability of our findings. Besides, our study design addresses critical gaps in the existing literature by distinguishing direct genetic effects from potential mediation by behavioral or environmental factors (a distinction observational studies cannot reliably make). These methodological and conceptual advances establish a foundation for future research to explore mechanistic pathways linking mental and metabolic health, with implications for targeted prevention strategies in high-risk PD populations.

## 5. Conclusions

We identified a statistically significant genetic association between OPD and coronary atherosclerosis (OR = 1.001, *P* = 0.029), suggesting shared biological pathways involving neurovascular dysfunction. No strong genetic evidence linking other PDs to CMDs was found, challenging previous observational associations and redirecting focus toward behavioral and environmental mediators. The results call for mechanistic studies integrating neuroimaging and vascular biomarkers to elucidate OPD-atherosclerosis pathways, and expanded genetic analyses using next-generation polygenic scores with improved PD coverage. Besides, it is necessary to target trials assessing cardiovascular risk reduction through behavioral modification in high-risk PD populations.

## Author contributions

**Conceptualization:** Wentao Yang.

**Funding acquisition:** Zhaoqing Yang.

**Resources:** Zian Cheng.

**Supervision:** Juemin Xi.

**Validation:** Cheng Bian.

## Supplementary Material


