# Supplementary material for: Personality disorders and cardiometabolic diseases: A Mendelian randomization study
Source: Medicine (Baltimore). 2026 Jan 9;105(2):e46702. doi: 10.1097/MD.0000000000046702 (PMC12795098; doi:10.1097/MD.0000000000046702)
Supplement: Supplementary file 1 [file medi-105-e46702-s001.docx]

**Supplementary Data 1: The download link of GWAS for PDs**

ANAPER:<http://storage.googleapis.com/finngen-public-data-r10/summary_stats/finngen_R10_F5_ANAPER.gz>

ANXPER:<http://storage.googleapis.com/finngen-public-data-r10/summary_stats/finngen_R10_F5_ANXPER.gz>

DEPPER:<http://storage.googleapis.com/finngen-public-data-r10/summary_stats/finngen_R10_F5_DEPPER.gz>

DISPER:<http://storage.googleapis.com/finngen-public-data-r10/summary_stats/finngen_R10_F5_DISPER.gz>

EMOPER:<http://storage.googleapis.com/finngen-public-data-r10/summary_stats/finngen_R10_F5_EMOPER.gz>

HISPER:<http://storage.googleapis.com/finngen-public-data-r10/summary_stats/finngen_R10_F5_HISPER.gz>

PARAPER:<http://storage.googleapis.com/finngen-public-data-r10/summary_stats/finngen_R10_F5_ANAPER.gz>

SCHIZPER:<http://storage.googleapis.com/finngen-public-data-r10/summary_stats/finngen_R10_F5_SCHIZPER.gz>

OPD:<http://storage.googleapis.com/finngen-public-data-r10/summary_stats/finngen_R10_F5_PERSBEHORG.gz>

**Supplementary Data 2: Code for selecting IVs**

R Code

1 setwd("E:/project/MR")

2

1. # Data version
2. version="R10"
3. # Names of exposures
4. exposure_array <- c(paste("finngen_",version,"_F5_ANAPER",sep=""),
5. paste("finngen_",version,"_F5_ANXPER",sep=""),
6. paste("finngen_",version,"_F5_DEPPER",sep=""),
7. paste("finngen_",version,"_F5_DISPER",sep=""),
8. paste("finngen_",version,"_F5_EMOPER",sep=""),
9. paste("finngen_",version,"_F5_HISPER",sep=""),
10. paste("finngen_",version,"_F5_PARAPER",sep=""),
11. paste("finngen_",version,"_F5_PERSBEHORG",sep=""),
12. paste("finngen_",version,"_F5_SCHIZPER",sep=""))
13. src_folder = ".extract_instruments_cached"
14. dst_folder = "exposure_F"

17

1. for (exposure_name in exposure_array) {
2. print(paste("Processing ",exposure_name,sep=""))
3. src_path = paste(src_folder,"/",exposure_name,".csv",sep="")

21

22 dir.create(dst_folder)

23 dst_path = paste(dst_folder,"/",exposure_name,".csv",sep="")

24

25 data <- read.csv(src_path)

26

27 # Calculate Fval

28 data <- data %>%

29 mutate(Fval = (beta.exposure/se.exposure)^2)

30

31 write.csv(data, file = dst_path, row.names = FALSE)

32 }

33

**Supplementary Data 3:** **The code for calculating IVW**

R Code

| 1 |  | |
| --- | --- | --- |
| 2 | # | Install following packages |
| 3 | # | install.packages("devtools") |
| 4 | # | devtools::install_github("huangyebao/ClumpMR") |
| 5 |  |  |

1. library(TwoSampleMR)
2. library(ClumpMR)

8

1. # Set as the path where this file is located
2. setwd("E:/project/MR")

11

1. # Set a temporary directory for VRoom decompression.
2. # This is set to the hard drive on my machine to avoid extensive writing to t he SSD drive
3. Sys.setenv(VROOM_TEMP_PATH = 'E:/project/MR/vroom_tmp')

15

1. # Repeat fn until successful execution. Execute max_times at most times.
2. retry_times <- function(fn, max_times) {
3. try_times <- 0
4. success <- FALSE
5. result <- NULL
6. while(success==FALSE && try_times < max_times){
7. if(try_times >= 1){
8. print(paste("Retry times: ", try_times))

24 }

25 tryCatch(

26 {

27 result = fn()

28 success <- TRUE

29

30 }, error = function(e) {

31 cat("Caught an error: ", conditionMessage(e), "\n")

32 return(NULL)

33 }

34 )

35 try_times <- try_times + 1

36 }

37 if(success==FALSE){

38 stop("Retry fail!")

39 }

40 return(result)

41 }

42

1. # Read exposure data from the {exposure_name}.gz file,
2. # filter it,
3. # modify the header,
4. # and eliminate linkage disequilibrium.
5. extract_exposure_from_local <- function(exposure_name, version){

48

1. # Read exposure data from the {exposure_name}.gz file
2. data <- vroom::vroom(paste("finngen_data/",version,"/",exposure_name,".gz", sep=""))

51

1. # Filter
2. exp_data <- subset(data,data$pval<5e-6)

54

1. # Filter & Modify the header
2. exposure_dat <- TwoSampleMR::format_data(
3. exp_data,
4. type = "exposure",
5. phenotype_col = "Phenotype",
6. snp_col = "rsids",
7. beta_col = "beta",
8. se_col = "sebeta",
9. eaf_col = "af_alt_cases",
10. effect_allele_col = "alt",
11. other_allele_col = "ref",
12. pval_col = "pval",
13. gene_col = "nearest_genes",
14. chr_col = "#chrom",
15. pos_col = "pos"

70 )

71

1. # Eliminate linkage disequilibrium online.
2. # Use api in https://github.com/huangyebao/ClumpMR，which internally calls the TwoSampleMR::clump_data function
3. exposure_dat <- clump_dat(exposure_dat, clump_kb = 10000, clump_r2 = 0.001, clump_p1 = 5e-6, clump_p2 = 5e-8, pop = "EUR")
4. return(exposure_dat)

76 }

77

1. # Provide caching functionality for the exposure table, so that the next time the same exposure table is needed, there is no need to recalculate
2. # Cached exposure tables are stored in .extract_instruments_cached folder.
3. # Warning: If you want to modify parameters in 'extract_exposure_from_local' and re-generate files, you must delete caches first.
4. extract_exposure_from_local_cached <- function(exposure_name, version){
5. file_path = paste(".extract_instruments_cached/",exposure_name,".csv",sep

="")

1. exposure <- NULL
2. if(file.exists(file_path)){

85

1. # If cache exists, read it.
2. print(paste("Read ",exposure_name," from cache: ",file_path))
3. exposure <- read.csv(file_path)

89

90 }else{

91

1. # If cache not exists, then generate and save it.
2. print(paste("Extract ",exposure_name," from .gz file"))
3. dir.create(".extract_instruments_cached")
4. exposure <- extract_exposure_from_local(exposure_name, version)
5. write.csv(exposure, file = file_path, row.names = FALSE)
6. print(paste("Save ",exposure_name," into cache: ",file_path))

98

99 }

100 return(exposure)

101 }

102

1. # Data version
2. version="R10"
3. # All names of exposures and outcomes.
4. exposure_array <- c(paste("finngen_",version,"_F5_ANAPER",sep=""),
5. paste("finngen_",version,"_F5_ANXPER",sep=""),
6. paste("finngen_",version,"_F5_DEPPER",sep=""),
7. paste("finngen_",version,"_F5_DISPER",sep=""),
8. paste("finngen_",version,"_F5_EMOPER",sep=""),
9. paste("finngen_",version,"_F5_HISPER",sep=""),
10. paste("finngen_",version,"_F5_PARAPER",sep=""),
11. paste("finngen_",version,"_F5_PERSBEHORG",sep=""),
12. paste("finngen_",version,"_F5_SCHIZPER",sep=""))
13. outcome_array <- c("ebi-a-GCST90038610",

116 "ebi-a-GCST90038689",

117 "ebi-a-GCST90018926",

118 "ebi-a-GCST90038604",

119 "ebi-a-GCST90038613",

1. "ukb-d-I9_CORATHER",
2. "ebi-a-GCST90018864",
3. "ebi-a-GCST90018890")
4. save_folder <- paste(".result_",version,sep="")
5. hete_save_folder <- paste(".heterogeneity_",version,sep="")
6. dir.create(save_folder)
7. dir.create(hete_save_folder)

127

1. # For each exposure
2. for (exposure_name in exposure_array) {
3. print(paste("Trying exposure = ", exposure_name))
4. exposure <- extract_exposure_from_local_cached(exposure_name, version)

132

1. # For each outcome
2. for (outcome_name in outcome_array) {

135

| 136 | # Prepare | save file name |
| --- | --- | --- |
| 137 | file_name | <- paste(exposure_name, outcome_name, "result",sep=" ") |
| 138 | file_name | = paste(save_folder,"/",file_name,".csv",sep="") |

1. hete_file_name = paste(exposure_name, outcome_name, "Q",sep=" ")
2. hete_file_name = paste(hete_save_folder,"/",hete_file_name,".csv",sep="")

141

1. # If the result has already been calculated, skip it.
2. # Application scenario: If the previous execution of this program exits m idway and some results have been saved locally, then this execution will directly sk ip these results to avoid duplicate calculations.

| 144 | if(file.exists(file_name)){ |
| --- | --- |
| 145 | print(paste(file_name," already exists.",sep="")) |
| 146 | next |
| 147 | } |
| 148 |  |
| 149 | # Finish the computation |
| 150 | tryCatch( |
| 151 | { |
| 152 | print(paste("Trying exposure = ", exposure_name," , outcome = ", outc |

ome_name,sep=""))

1. outcome <- retry_times(function() {extract_outcome_data(snps=exposure

$SNP,outcomes=outcome_name)}, 10)

1. print(paste("Get outcome data: ",outcome_name,sep=""))
2. dat <- harmonise_data(exposure, outcome)
3. res_hete <- mr_heterogeneity(dat)

157

1. mrResult <- mr(dat)
2. mrResult <- generate_odds_ratios(mrResult)

160

1. # save 'myResult' table to .csv file
2. write.csv(mrResult, file = file_name, row.names = FALSE)
3. write.csv(res_hete, file = hete_file_name, row.names = FALSE)
4. print(paste(file_name," save success!"))

| 165 |  | }, error = function(e) { |
| --- | --- | --- |
| 166 |  | cat("Skip ",file_name,". Caught an error: ", conditionMessage(e), |
| "\n")  167 |  | } |
| 168 |  | ) |
| 169 |  |  |
| 170 |  |  |
| 171  172 } | } |  |
| 173 |  |  |
| 174 |  |  |
| 175 |  |  |

**Supplementary Data 4: Code for calculating the or-value and ci-value**

R Code

1. library(TwoSampleMR)
2. library(ClumpMR)

3

1. # Calculate or and ci in each mrresult
2. # TODO: merge this process into mr_local.R

6

1. setwd("E:/project/MR")
2. read_folder_path <- ".result_local_R10"
3. write_folder_path <- ".result_local_R10_ci"
4. dir.create(write_folder_path)
5. file_list <- list.files(read_folder_path, full.names = FALSE, recursive = FAL SE)

12

1. for (file in file_list) {
2. print(file)
3. read_file_path = file.path(read_folder_path, file)
4. write_file_path = file.path(write_folder_path, file)
5. # Deal each file
6. mr_results = read.csv(read_file_path)
7. mr_results_with_or_ci <- generate_odds_ratios(mr_results)
8. write.csv(mr_results_with_or_ci, file = write_file_path, row.names = FALSE)

21 }

22

23

**Supplementary Data 5: Code for extracting the result of IVW and MR Egger**

**import** os

**import** math

**def extrace_data**(file_path, extract_fn):

**with** open(file_path, 'r') **as** f: lines = f.readlines() ivw_data = None

mr_egger = None

**for** line **in** lines:

**if** "Inverse variance weighted" **in** line: ivw_data = extract_fn(line)

**elif** "MR Egger" **in** line: mr_egger = extract_fn(line)

*# pval = ivw_line.split(',')[col_idx].strip()*

**return** {"ivw_data":ivw_data, "mr_egger":mr_egger}

**def extract_all_data**(folder_path, extract_fn): result = dict()

**for** dirpath, dirnames, filenames **in** os.walk(folder_path):

**print**(len(filenames))

**for** filename **in** filenames:

file_path = os.path.join(dirpath, filename) data = extrace_data(file_path, extract_fn) exposure_name = filename.split(' ')[0] outcome_name = filename.split(' ')[1] result[(exposure_name, outcome_name)] = data

**return** result

**def extract_all_data_to_csv**(folder_path, save_path, exposure_names, outcome_names, extract_fn): result = extract_all_data(folder_path, extract_fn)

**with** open(save_path, 'w') **as** f: f.write("-,")

f.write(",".join(outcome_names) + '\n')

**for** exposure_name **in** exposure_names: f.write(exposure_name)

**for** outcome_name **in** outcome_names:

data = result.get((exposure_name, outcome_name), 'NA') f.write(',' + pval)

f.write('\n')

**def flatten_extract**(folder_path, save_path, exposure_names, outcome_names, outcome_name_map):

**def extract_b_se_pval**(line): items = line.split(',')

b = float(items[6].strip()) se = float(items[7].strip())

pval = float(items[8].strip()) fval = (b / se) * (b / se) or_ = float(items[11].strip())

or_lci95 = float(items[12].strip()) or_uci95 = float(items[13].strip()) *# ro = math.exp(b)*

**return** str(b),str(se),str(pval), str(fval), str(or_), str(or_lci95), str(or_uci95) val_num = 9

result = extract_all_data(folder_path, extract_b_se_pval) os.makedirs(save_path, exist_ok=True)

**for** exposure_name **in** exposure_names:

save_file_path = os.path.join(save_path, f"{exposure_name}.csv")

**with** open(save_file_path, 'w') **as** f: f.write("outcome_db,outcome,b,se,pval,F=(b/se)^2,or,or_lci95,or_uci95\n")

**for** outcome_db_name **in** outcome_names:

outcome_name = outcome_name_map[outcome_db_name] f.write(f"{outcome_db_name},{outcome_name},,,,,,,\n") **assert** (exposure_name, outcome_db_name) **in** result data = result.get((exposure_name, outcome_db_name)) data_mr_egger = data["mr_egger"]

data_ivw = data["ivw_data"]

**if** data_mr_egger **is** None: data_mr_egger = ["NA"] * val_num

**if** 'NA' **not in** data_mr_egger:

f.write(f",MR Egger,{','.join(data_mr_egger)}\n") f.write(f",Inverse variance weighted,{','.join(data_ivw)}\n")

version = "R10"

**def extract_or**(line):

b = float(line.split(',')[-3].strip()) ro = math.exp(b)

**return** str(ro)

**def extrace_pval**(line):

pval = line.split(',')[-1].strip()

**return** pval

folder_path = f'.result_local_{version}_ci' save_path = f'.result_local_{version}_flatten' outcome_names = [

"ebi-a-GCST90038610", "ebi-a-GCST90038689", "ebi-a-GCST90018926", "ebi-a-GCST90038604", "ebi-a-GCST90038613", "ukb-d-I9_CORATHER", "ebi-a-GCST90018864", "ebi-a-GCST90018890"

]

outcome_name_map = {

"ebi-a-GCST90038610":"Myocardial infarction", "ebi-a-GCST90038689":"Atrial fibrillation", "ebi-a-GCST90018926":"Type 2 diabetes",

"ebi-a-GCST90038604":"Hypertention", "ebi-a-GCST90038613":"Stroke",

"ukb-d-I9_CORATHER":"Coronary atherosclerosis", "ebi-a-GCST90018864":"Ischemic stroke",

"ebi-a-GCST90018890":"Peripheral artery disease"

}

exposure_names = [ f"finngen_{version}_F5_ANAPER", f"finngen_{version}_F5_ANXPER", f"finngen_{version}_F5_DEPPER", f"finngen_{version}_F5_DISPER", f"finngen_{version}_F5_EMOPER", f"finngen_{version}_F5_HISPER", f"finngen_{version}_F5_PARAPER", f"finngen_{version}_F5_PERSBEHORG", f"finngen_{version}_F5_SCHIZPER"

]

*# extract_all_data_to_csv(folder_path, save_path, exposure_names, outcome_names, extract_fn=extrace_pval)*

flatten_extract(folder_path, save_path, exposure_names, outcome_names, outcome_name_map)

**print**("Down")

**Supplementary Data 6: Code for drawing forest plots**

R Code

1 install.packages("forestploter")

2

3 {

1. library(TwoSampleMR)
2. library(ggplot2)
3. library(dplyr)
4. library(gridExtra)
5. library(grid)

9 }

1. library(forestploter)
2. # Setting the theme
3. tm <- forest_theme(
4. base_size = 15,
5. ci_pch = 21,
6. ci_col = "grey20",
7. ci_fill = "red",
8. ci_alpha = 1,
9. ci_lwd = 1,
10. ci_Theight = 0.5,

20

1. refline_lwd = 1,
2. refline_lty = "dashed",
3. refline_col = "grey20",

24

1. vertline_lwd = 2,
2. vertline_col = "grey20",

27

1. footnote_cex = 0.6,
2. footnote_fontface = "italic",
3. footnote_col = "red4",

31

1. arrow_lwd = 1,
2. arrow_fill = "red",
3. arrow_col = "red"

35 )

36

37

1. draw <- function(exposure_file_name, exposure_name){
2. data <- read.csv(paste(".result_local_R10_flatten/finngen_R10_F5_",exposure

_file_name,".csv",sep="")) # 替换为实际路径

40

1. # Title
2. condition <- is.na(data$or)
3. row_indices <- which(condition)

44

1. # Create
2. data$outcome <- ifelse(is.na(data$or),
3. data$outcome,
4. paste0(" ", data$outcome))
5. data$pval <- ifelse(is.na(data$pval), "", sprintf("%.2f",data$pval))

50

51 # Create blank columns for drawing forest plot

| 52 | data$` ` <- paste(rep(" ", 24), collapse = " ") # col 10 |
| --- | --- |
| 53 | # Create confidence interval columns for display |
| 54 | data$`OR (95% CI)` <- ifelse(is.na(data$or), "", |
| 55 | sprintf("%.4f (%.4f, %.4f)", |
| 56 | data$or, data$or_lci95, data$or_uci9 |
| 5)) # col | 11 |
| 57 | # padding |
| 58 | #data$outcome <- paste0(data$outcome, " ") |
| 59 | #data$pval <- paste0(data$pval, " ") |
| 60 | #data$`OR (95% CI)` <- paste0("",data$`OR (95% CI)`, " ") |
| 61 |  |
| 62 | # Padding between columns |
| 63 | data$` ` <- paste(rep(" ", 4), collapse = " ") # col 12 |
| 64 | data$` ` <- paste(rep(" ", 4), collapse = " ") # col 13 |
| 65 | data$` ` <- paste(rep(" ", 4), collapse = " ") # col 14 |
| 66 |  |
| 67 | # Draw forest plot |
| 68 | p <- forest(data[,c(2, 12, 5, 13, 10, 14, 11)], # Select the columns of d |

ata to be used in the forest plot

69 est = data$or, # The effect size, which is the HR column

70 lower = data$or_lci95, # The lower limit of the confidence inter val

71 upper = data$or_uci95, # The upper limit of the confidence inter val

72 sizes = 1, # The size of the black squares

73 ci_column = 5, # Plot the forest plot in the 3rd column (confidence interval column)

| 74 | ref_line = 1, | # | Add |
| --- | --- | --- | --- |
| 75 | xlim = c(0.5, 1.5), | # | Set |
| 76 | ticks_at = c(0.5, 1, | 1.5), # | |
| 77 | xlab="Odds Ratio", |  | |

a reference line

the range of the x-axis

Add ticks at specified positions

| 78 | theme = tm, | | # Add a custom theme |
| --- | --- | --- | --- |
| 79 | ) | |  |
| 80  81 | # Setting colors | |  |
| 82 | for (row | in 1:nrow(data)){ | |
| 83 | if(row | %in% row_indices){ | |
| 84 | p <- | edit_plot(p, row = row, which = "background", | |
| 85 |  | gp = gpar(fill = "#F6F6F6")) | |
| 86 | }else{ |  | |
| 87 | p <- | edit_plot(p, row = row, which = "background", | |
| 88 |  | gp = gpar(fill = "#ffffff")) | |
| 89 | } | | |
| 90 | } | |  |
| 91 |  | |  |
| 92 | p <- insert_text(p, | |  |
| 93 | text | | = exposure_name, |
| 94 |  | col = 1:7, | |

95 part = "header",

96 gp = gpar(fontface = "bold", fontsize=15))

97

98 my_plot = plot(p)

99

100 ggsave(paste(".forest_plot/",exposure_file_name,".pdf",sep=""),my_plot,widt

h = 12, height = 8)

| 101 | } |  |
| --- | --- | --- |
| 102 |  |  |
| 103 | setwd("E:/project/MR") |  |
| 104 | exposures = list( |  |
| 105 | list("ANAPER", "Anankastic personality disorder"), |  |
| 106 | list("ANXPER", "Anxious personality disorder"), |  |
| 107 | list("DEPPER","Dependent personality disorder"), |  |
| 108 | list("DISPER","Dissocial personality disorder"), |  |
| 109 | list("EMOPER","Emotionally unstable personality disorder"), |  |
| 110 | list("HISPER","Histrionic personality disorder"), |  |
| 111 | list("PARAPER","Paranoid personality disorder"), |  |
| 112 | # PERSBEHORG: Personality and behavioural disorders due to brain | disease, d |
| amage | and dysfunction |  |
| 113 | # OPD: Organic Personality Disorder |  |
| 114 | # PERSBEHORG == OPD |  |
| 115 | list("PERSBEHORG","Organic personality disorder"), |  |
| 116 | list("SCHIZPER","Schizoid personality disorder") |  |
| 117 | ) |  |
| 118 | for (i in 1:length(exposures)) { |  |
| 119 | sublist <- exposures[[i]] |  |
| 120 | exposure_file_name = sublist[[1]] |  |
| 121 | exposure_name = sublist[[2]] |  |
| 122 | print(paste("Processing",exposure_file_name,"...")) |  |
| 123 | draw(exposure_file_name, exposure_name) |  |
| 124 | } |  |
| 125 |  |  |
| 126 |  |  |

**Supplementary Data 7: Code for generating leave-one-out analysis**

R Code

1

1. library(TwoSampleMR)
2. library(ClumpMR)
3. library(dplyr)

5

1. # Set as the path where this file is located
2. setwd("E:/project/MR")

8

1. # Set a temporary directory for VRoom decompression.
2. # This is set to the hard drive on my machine to avoid extensive writing to t he SSD drive
3. Sys.setenv(VROOM_TEMP_PATH = 'E:/project/MR/vroom_tmp')

12

13

1. do_the_leaveoneout_plot <- function(exposure_name, outcome_name, exposure_sho rt_name){
2. print(exposure_name)
3. print(outcome_name)
4. file_path = paste(".extract_instruments_cached/",exposure_name,".csv",sep

="")

1. exposure <- read.csv(file_path)
2. outcome <- extract_outcome_data(snps=exposure$SNP,outcomes=outcome_name)
3. dat <- harmonise_data(exposure, outcome)
4. single <- mr_leaveoneout(dat)

22

23 # change exposure's name

24 single <- single %>%

25 mutate(exposure = exposure_short_name)

26

27 plot <- mr_leaveoneout_plot(single)

28

29 # Assign tables to global environment

30 assign("exposure", exposure, envir = .GlobalEnv)

31 assign("outcome", outcome, envir = .GlobalEnv)

32 assign("dat", dat, envir = .GlobalEnv)

33 assign("single", single, envir = .GlobalEnv)

34 return(plot)

35 }

36

37 do_the_leaveoneout_plot("finngen_R10_F5_PERSBEHORG", "ukb-d-I9_CORATHER", "PE RSBEHORG")

38

**Supplementary Data 8: Code foe generating Cochra’s Q test**

R Code

1. setwd("E:/project/MR")
2. hete_save_folder <- paste(".heterogeneity_",version,sep="")
3. save_path="heterogeneity.csv"

4

5 all_hete_data = NULL

6

1. for (exposure_db_name in exposure_array) {
2. exposure_name = exposure_name_map[[exposure_db_name]]

9

1. for (outcome_db_name in outcome_array) {
2. print(paste("Processing exposure:",exposure_db_name,"outcome:",outcome_db

_name,sep=" "))

12

13 outcome_name = outcome_name_map[[outcome_db_name]]

14

1. hete_file_name = paste(exposure_db_name, outcome_db_name, "Q",sep=" ")
2. hete_file_name = paste(hete_save_folder,"/",hete_file_name,".csv",sep="")
3. hete_data = read.csv(hete_file_name)
4. hete_data$"exposure_db_name" = exposure_db_name
5. hete_data$"exposure_name" = exposure_name
6. hete_data$"outcome_db_name" = outcome_db_name
7. hete_data$"outcome_name" = outcome_name
8. hete_data <- hete_data[, c("id.exposure", "id.outcome", "outcome", "expos ure", "exposure_db_name", "exposure_name", "outcome_db_name", "outcome_name", "metho d", "Q","Q_df","Q_pval")]

| 23 |  | if(is.null(all_hete_data)){ |
| --- | --- | --- |
| 24 |  | all_hete_data = hete_data |
| 25 |  | }else{ |
| 26 |  | all_hete_data = rbind(all_hete_data, hete_data) |
| 27 |  | } |
| 28  29 } | } |  |

30 write.csv(all_hete_data, file = save_path, row.names = FALSE)

31
